# Does creatine affect lipid profile? a systematic review and meta-analysis of randomized placebo-controlled trials

**DOI:** 10.3389/fnut.2026.1787009

**Published:** 2026-05-08

**Authors:** Fabiana V. M. Gimenez, Natalia Pasternak, Lívia Santesso, Lívia M. A. da Silva, Lara A. Girotto, Bruno M. Candeloro, Sandra Maria Barbalho, Andrey A. Porto, Rodrigo D. Raimundo, Luiz Carlos de Abreu, Vitor E. Valenti

**Affiliations:** 1 Systematic Reviews Center for Cardiovascular and Metabolic Health, School of Philosophy and Sciences, São Paulo State University (UNESP), São Paulo, Brazil; 2 Professor of the Nursing Course and the Postgraduate Program in Health and Aging, Faculdade de Medicina de Marília (FAMEMA), São Paulo, Brazil; 3 Columbia University, New York, NY, United States; 4 Department of Biochemistry and Pharmacology, School of Medicine, Universidade de Marília (UNIMAR), Marília, São Paulo, Brazil; 5 Department of Biochemistry and Nutrition, School of Food and Technology of Marília (FATEC), São Paulo, Brazil; 6 Research Coordinator, UNIMAR Charity Hospital, Universidade de Marília (UNIMAR), Marília, São Paulo, Brazil; 7 Laboratório de Delineamento de Estudos e Escrita Científica, Centro Universitário FMABC, Santo André, Brazil; 8 University of Limerick, Limerick, Ireland; 9 Federal University of Espirito Santo, Vitoria, Brazil

**Keywords:** cholesterol, creatine, HDL, LDL, lipid profile

## Abstract

**Introduction:**

Creatine, recognized for its regulatory functions in various metabolic tissues, has aroused interest regarding its systemic effects. This study aimed to evaluate the impact of creatine supplementation on blood lipid profile parameters.

**Methods:**

A systematic review with meta-analysis was conducted following the PRISMA guidelines (CRD420251025690). Searches were conducted in EMBASE, Lilacs, CINAHL, MEDLINE/PubMed, Cochrane, Scopus, and Web of Science databases up to June 2025. Randomized clinical trials involving adults receiving oral creatine supplementation versus placebo were included. The outcomes analyzed included triglycerides, total cholesterol, low density lipoprotein cholesterol (LDL-C), and high density lipoprotein cholesterol (HDL-c). Risk of bias was assessed using the Cochrane RoB 2 tool, and certainty of evidence was assessed using the GRADE system.

**Results:**

We selected 8 RCTs. Pooled analyses demonstrated no statistically significant effects of creatine supplementation on total cholesterol (MD: 2.9 mg/dL; 95% CI: −7.44 to 13.24; *I^2^* = 46%), LDL-C cholesterol (MD: 4.08 mg/dL; 95% CI: −2.55 to 10.70; *I^2^* = 8%), HDL-C (MD: −0.68 mg/dL; 95% CI: −3.94 to 2.59; *I^2^* = 0%), or triglycerides (MD: 7.95 mg/dL; 95% CI: −13.73 to 29.63; *I^2^* = 55%). The certainty of evidence was classified as very low for total cholesterol and triglycerides and low for LDL-C and HDL-C. Methodological limitations were identified in the randomization and reporting of results processes.

**Conclusion:**

Creatine supplementation did not demonstrate clinically relevant effects on lipid profiles. Future studies with greater methodological rigor and larger samples are recommended for definitive confirmation of these findings.

## Introduction

Dietary and sports supplements have become increasingly popular not only among high-performance athletes but also in the general population. This rapid expansion—estimated at USD 192.65 billion in 2024 and projected to reach USD 414.52 billion by 2033 Grand View Research (1)—has been accompanied by widespread misconceptions, ranging from exaggerated performance claims to unfounded safety concerns. The scientific community has responded with growing scrutiny of extraordinary health and weight-loss promises that lack robust evidence, reinforcing the need for critical appraisal and public clarification regarding risks and benefits (2).

Creatine occupies a unique position within this landscape. Endogenously synthesized in the liver, kidneys, and pancreas, creatine is highly concentrated in tissues with elevated energy demands. Its primary physiological role involves cellular energy buffering through the phosphocreatine system, catalyzed by creatine kinase (3, 4). Despite strong evidence supporting its ergogenic effects, misconceptions persist, including beliefs that creatine causes excessive water retention, kidney damage, baldness, muscle cramping, dehydration, steroid-like effects, or fat gain (5).

Beyond skeletal muscle metabolism, emerging research suggests broader systemic roles. Experimental studies indicate that creatine may influence thermogenesis and mitochondrial ATP turnover in adipose tissue, potentially increasing energy expenditure and diet-induced thermogenesis (6). Additionally, creatine has been described as possessing antioxidant properties and membrane-stabilizing effects, suggesting potential relevance in oxidative stress and inflammatory contexts (4, 7). These mechanistic findings have led to hypotheses linking creatine supplementation to lipid metabolism.

However, human evidence regarding creatine’s effects on blood lipid profiles remains limited and inconsistent. Clarke et al. (8) recently reported improvements in triglyceride levels following supplementation. In contrast, Gualano et al. (9) found no additional lipid changes when creatine was combined with cardiorespiratory training compared to exercise alone. Similarly, Almeida et al. (34) and Arciero et al. (10) observed no significant effects on total cholesterol, low density lipoprotein cholesterol (LDL-C), high density lipoprotein cholesterol (HDL-C), or triglycerides. Thus, despite biologically plausible mechanisms, it remains uncertain whether creatine exerts clinically meaningful effects on plasma lipids in humans.

This question is particularly relevant given the central role of dyslipidemia in cardiometabolic risk. Elevated LDL-C, triglycerides, and total cholesterol, along with reduced HDL-C, are established contributors to cardiovascular disease (11, 12). LDL-C and non-HDL-C are especially strong predictors of cardiovascular events, particularly among individuals with obesity, advanced age, or suboptimal dietary patterns (12). Consequently, identifying safe, adjunctive strategies to favorably modulate lipid profiles remains a clinical priority.

Systematic reviews and meta-analyses provide an essential framework for synthesizing heterogeneous findings, quantifying pooled effects, and identifying sources of variability. By applying rigorous methodologies such as PRISMA and GRADE (13, 14), evidence synthesis can generate clinically meaningful conclusions and guide future research. Therefore, we conducted a systematic review and meta-analysis of randomized placebo-controlled trials to clarify the effect of creatine supplementation on blood lipid levels.

## Methods

### Protocol and registration

The review was conducted following the guidelines of the Preferred Reporting Items for Systematic Reviews and Meta-Analyses (PRISMA) (14) and has been registered in the PROSPERO database (CRD420251025690).

### Eligibility criteria

The studies were sourced from peer-reviewed journals, spanning from the inception of each database through March 2026. Eligibility was determined based on predefined inclusion and exclusion criteria aligned with the PICOS framework (Population, Intervention, Comparison, Outcomes, and Study Design), which encompassed:

(P) Adults aged 18 years and older, regardless of sex or ethnicity, apparently healthy individuals or those with metabolic risk factors (…) were included if the lipid profile was reported as an outcome. We excluded studies involving children or adolescents (<18 years) and animal studies or *in vitro* research;
(I) Oral creatine supplementation (any form: monohydrate, ethyl ester, etc.), administered either alone or in combination with exercise, provided that the creatine effect can be isolated or quantified. We excluded studies that combined creatine with other supplements without isolating its effect;
(C) For comparison groups, we included studies that evaluated subjects that received placebo;
(O) Studies reporting at least one lipid-related outcome, such as total cholesterol, low-density lipoprotein cholesterol (LDL-C), high-density lipoprotein cholesterol (HDL-C), triglycerides (TG) and other markers of lipid metabolism (e.g., apolipoproteins, non-HDL-C);
(S) We included single or double-blind randomized controlled trials as well as crossover designs. This review was limited to studies published in peer-reviewed journals, along with master’s theses and doctoral dissertations. Conference abstracts, descriptive studies, case reports, editorials, and review articles were excluded.

### Information source, search strategy and study selection

The searches were conducted in EMBASE, LILACS, CINAHL, MEDLINE/PubMed (via the National Library of Medicine), Cochrane, Scopus, and Web of Science using the following keywords: “Creatine Supplement” OR “Creatine monohydrate supplementation” OR “Creatine supplementation” AND “Cholesterol” OR “Epicholesterol” AND “Triacylglycerol” OR “Triglyceride” OR “HDL” OR “LDL” (Supplementary file). All retrieved records were exported to the Rayyan QCRI platform (Qatar Computing Research Institute, Qatar) for duplicate removal. Screening was then carried out in Rayyan by assessing titles and abstracts. Full-text eligibility was independently evaluated by at least two reviewers, with a third reviewer consulted in cases of disagreement. After finalizing the included studies, the research team collectively assessed the feasibility of conducting a meta-analysis.

### Data collection and data extraction

Data regarding authorship, study design, participant characteristics, intervention protocols, comparator details, and outcome measures were extracted from the primary studies and summarized in the Table. When required information was unclear or incomplete, attempts were made to contact the corresponding authors. Data extraction was performed independently by at least two reviewers, and discrepancies were resolved by consensus.

For studies reporting lipid outcomes at multiple time points (e.g., Earnest et al., which assessed outcomes at 4 and 8 weeks; Gualano et al., which evaluated outcomes at weeks 4, 8, and 12), we pre-specified the time point corresponding to the end of the intervention period as the primary analysis point. When multiple interim assessments were reported, only the final available measurement under supplementation was included in the meta-analysis to avoid unit-of-analysis errors and selective inclusion of time points. This decision was defined *a priori* and applied consistently across all eligible studies, in accordance with PRISMA Items 9 and 10a (14).

If no response was obtained from authors and relevant numerical data were presented only graphically, values were extracted using WebPlotDigitizer®. All outcomes were expressed as means with standard deviations (SD). When primary studies reported standard errors (SE) or confidence intervals (CI), these were converted to SD using established statistical formulas to ensure consistency in pooled analyses.

### Data items

We collected data on cholesterol, LDL-C, HDL-C, triglycerides, and total lipid levels to compare outcomes following the intervention. Additional information on participant characteristics, intervention details, and funding sources was extracted from the included studies. Any missing or unclear data were excluded from the analysis.

### Assessment of the risk of bias in individual studies and across studies

The risk of bias assessment was conducted using the Cochrane Risk of Bias tool (15) within the Review Manager software (RevMan 5.4.1). This tool evaluates bias across six domains: “Randomization process,” “Deviations from intended interventions,” “Missing outcome data,” “Measurement of the outcome,” “Selection of the reported results,” and “Overall bias.” Each domain was rated as presenting a low risk, some concerns, or a high risk of bias, based on the criteria outlined in Sterne et al. (15) and the accompanying guidance for reviewers’ judgments. Two independent reviewers carried out the assessment, with a third researcher consulted in cases of disagreement.

Potential sources of bias affecting the cumulative evidence, such as publication bias and selective reporting, were also examined. All assessors underwent appropriate training sessions prior to performing the risk of bias evaluation.

### Certainty assessment (levels of evidence)

We applied the Grades of Recommendation, Assessment, Development, and Evaluation (GRADE) approach (GRADE Working Group, 2004) to assess the certainty of the evidence. This framework considers randomized trial design as the initial indicator of high-quality evidence, while also accounting for study quality, methodological rigor, and potential limitations that may reduce confidence in the findings (13). The Summary of Findings table was generated using GRADEpro GDT v4® (McMaster University, Ontario, Canada).

### Qualitative analysis (systematic review)

A narrative synthesis was conducted to provide detailed descriptions of how each study was carried out. Study-specific information was presented in both text and tables. The qualitative results of individual studies were assessed by examining cardiovascular parameters across intervention and control protocols.Synthesis of results and summary measures

After finalizing the selection of references, we evaluated the feasibility of conducting a meta-analysis. When feasible, outcome values were extracted, focusing on post-intervention data. All available post-intervention comparisons between groups were included. Heterogeneity was assessed using the I^2^ statistic, interpreted as follows: 0–29% = not important; 30–49% = moderate; 50–74% = substantial; and 75–100% = considerable heterogeneity (16, 17). For overall effect estimates and 95% confidence intervals (CI), statistical significance was defined as *p* < 0.05. When studies did not report measures of variability (e.g., SD, 95% CI, SE, or *p*-values), missing standard deviations of change scores (SDchanges) were calculated.

Meta-analytic outcomes were expressed as weighted mean differences (MD) with 95% confidence intervals (CI) and corresponding *p*-values when studies reported lipid outcomes using the same units and measurement scales (e.g., mg/dL). When outcomes were reported using different scales or units across studies, pooled effects were calculated using standardized mean differences (SMD) with 95% CI and p-values to ensure comparability across trials. Statistical significance was set at *p* < 0.05 for overall MD comparisons between intervention and control groups. Results were displayed in forest plots. A random-effects model was applied, as it is a more conservative approach that allows heterogeneity to extend beyond chance and enhances generalizability (18). Model selection considered both statistical heterogeneity and clinical/methodological diversity across studies. In the absence of important heterogeneity, a fixed-effect model was applied. When substantial heterogeneity was detected and clinical diversity was present, a random-effects model was used to account for between-study variability. Results were presented using forest plots. All analyses were conducted using Review Manager (RevMan 5.4.1).

## Results

### Study selection

**Figure 1:**
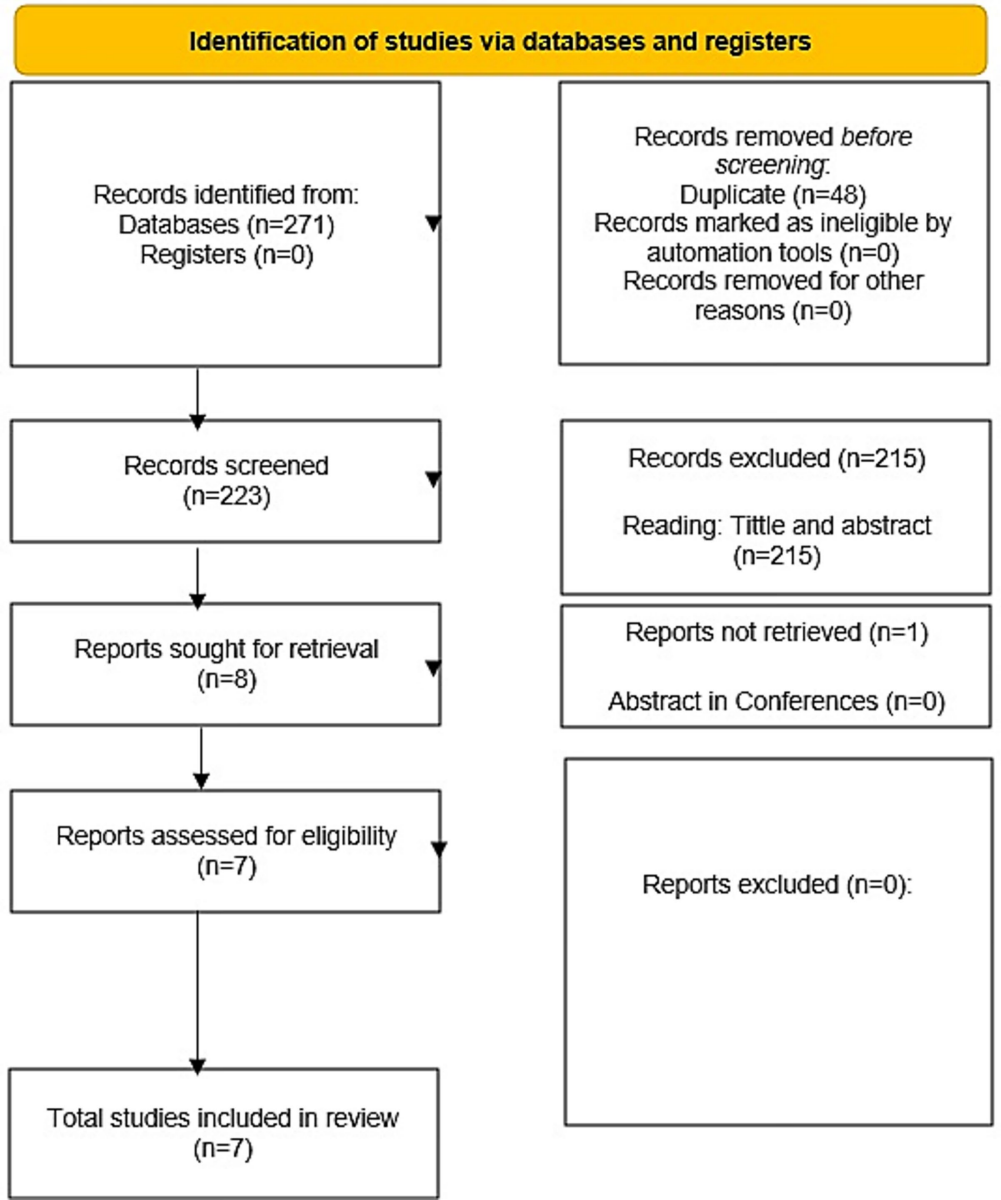
PRISMA 2020 flow diagram for new systematic reviews which included searches of databases and registers only.

A total of 271 references were identified through database searches. After removing 48 duplicates, 223 unique references were screened according to the inclusion criteria. Following the screening of titles and abstracts, 215 records were excluded. Eight studies were then sought for retrieval and full-text assessment. Consequently, eight reports were assessed for eligibility, leading to the final inclusion of studies in the review. The search methods and study selection process were conducted in accordance with the PRISMA statement, as illustrated in Figure 1. Detailed information on the included studies, including study design, sample size, intervention, control, main results, and funding, is provided in Table 1.

**Table 1:** Description of the characteristics of the study population of articles by author and year, sample, age (years), intervention, control, outcomes and funding.

Author/years	Study design	Sample	Age (years)	Intervention	Control	Outcomes	Funding
Almeida et al. (33)	Randomized, double-blind, placebo-controlled trial.	18 male resistance-training practitioners creatine group: *n* = 9 Control group: *n* = 9	Control group: 24.2 ± 3.7 years creatine group: 22.7 ± 3.0 years	Creatine monohydrate was administered at 0.3 g/kg/day for 7 days, divided into four equal doses per day, taken at: breakfast (7–8 a.m.) lunch (12 a.m.–1 p.m.) evening (4–5 p.m.) dinner (8–9 p.m.)	The control group received dextrosol as the placebo/control supplement.	No significant changes in total cholesterol, LDL-C, HLD-C and triglycerides	Not informed
Almeida et al. (34)	Randomized, double-blind, placebo-controlled trial.	Total participants: 34 healthy males creatine group (CREA): *n* = 17 Placebo group (PLA): *n* = 17	26.7 ± 4.8 years.	Dose: loading phase: 20 g/day of creatine monohydrate for 7 days. Maintenance phase: 5 g/day for 20 days. Total duration: 27 days of supplementation.	Maltodextrin in the same dose and duration as the creatine group, matching color and flavor.	TC ↑ at day 7 in both groups. LDL-C ↑ transiently in CREA, but was lower vs. PLA at day 7. HDL-C showed no between-group difference, with slight ↑ in PLA at day 30. TG ↑ only transiently in CREA at day 7, with no sustained effect.	No.
Arciero et al. (10)	Double-blind, randomized fashion, placebo-controlled design.	Total participants: 30 healthy males creatine only: *n* = 10 creatine + resistance training: *n* = 10 Placebo + resistance training: *n* = 10	21 ± 3 years.	Dose: loading phase: 20 g/day of creatine monohydrate for 5 days. Maintenance phase: 10 g/day for 23 days. Total duration: 28 days of supplementation.	The P-RT (dextrose) group ingested the placebo drink 4 times daily for 5 days and twice daily for 23 days.	TC ↑ in both groups at day 7. LDL-C ↑ transiently in CREA, but was lower vs. PLA at day 7. HDL-C showed no significant between-group difference. TG ↑ only transiently in CREA, with no sustained elevation and no significant between-group difference.	No.
Clarke et al. (8)	Randomized, double-blind, placebo-controlled, crossover design.	Total participants: 12 sedentary older adults creatine phase: *n* = 12 Placebo phase: *n* = 12	58.3 ± 3.4 years.	Dose: loading phase: 20 g/day of creatine monohydrate for 5 days. Maintenance phase: 5 g/day for 23 days. Total duration: 28 days of supplementation.	Both CrM and PL (maltodextrin) supplementation were consumed following the same dosing protocol: 4 × 5 g/day for 5 days, followed by 1 × 5 g/day for 23 days.	TTG ↓ significantly with CREA, while no change was observed with PLA. TC, LDL-C, and HDL-C showed no significant effects of CREA.	Yes.
Cornelissen et al. (19)	Single centre double-blind randomized placebo controlled trial.	Total participants: 70 cardiac patients creatine group: *n* = 33 Placebo group: *n* = 37	57.5 ± 8.4 years	Dose: loading phase: 15 g/day (3 × 5 g/day) for 7 days. Maintenance phase: 5 g/day for approximately 11 weeks. Total duration: 3 months of supplementation.	The placebo mixture contained 8.5 g maltodextrine, completed with starch and sweetener.	TG ↓ significantly with CREA, while no change was observed with PLA. TC, LDL-C, and HDL-C showed no significant effects of CREA.	Yes.
Earnest et al. (20)	A randomized, double-blind, placebo-controlled trial utilizing creatine as a potential lipid-lowering agent was conducted to determine plasma lipid, lipoprotein, glucose, urea nitrogen and creatinine profiles	Total participants: 19 moderately hypercholesterolemic males creatine group: *n* = 10 Placebo group: *n* = 9	50.9 ± 3.2 years	Dose: loading phase: 20 g/day of creatine monohydrate for 5 days. Maintenance phase: 10 g/day for 51 days. Total duration: 56 days (8 weeks) of supplementation.	Glucose placebo (6 g of glucose) for 56 days. creatine and placebo were taken orally four times a day for 5 days and then twice a day for 51 days.	Total cholesterol ↓ 6% at week 4 ↓ 5% at week 8 triglycerides ↓ 23% at week 4 ↓ 22% at week 8 ↓ 26% at week 12 significant reductions compared to placebo (*p* < 0.01) LDL No significant changes HDL No significant changes	Yes.
Gualano et al. (9)	A 12-wk, double-blind, randomized, placebo-controlled trial was conducted between 1 March and 1 June 2006.	Total participants: 22 sedentary healthy males creatine group: *n* = 12 Placebo group: *n* = 10	18–35 years old	Dose: loading phase: ~0.3 g/kg/day (~20 g/day) for 7 days. maintenance phase: ~0.15 g/kg/day (~10 g/day) for 11 weeks. Total duration: 12 weeks of supplementation.	The PL group was given dextrose in place of Cr, at the same dose.	TC: no significant change with CREA. LDL-C: no significant CREA vs. PLA difference. HDL-C: ↑ over time in both groups (training effect), with no additional effect of CREA. TG: ↓ over time in both groups (training effect).	Yes.
Volek et al. 2000(21)	n randomly assigned in a double-blind fashion to either a creatine	Total participants: 19 resistance-trained males creatine group: *n* = 10 Placebo group: *n* = 9	Age, 25_ + 4.8 and 25.4_ + 5.9 years	Dose: loading phase: 25 g/day for 7 days. maintenance phase: 5 g/day for 11 weeks. total duration: 12 weeks of supplementation.	While the placebo group ingested an equal number of placebo capsules.	Total cholesterol: No significant change compared to placebo remained within normal clinical ranges HDL: No significant effect LDL: No significant change Triglycerides: No significant change	No.

CREA, Creatine group; PLA, Placebo group; CrM, Creatine monohydrate; PL, Placebo; TC, Total cholesterol; LDL-C, Low-density lipoprotein cholesterol; HDL-C, High-density lipoprotein cholesterol; TG, Triglycerides; MD, Mean difference; RT, Resistance training; P-RT, Placebo plus resistance training; g/day, grams per day; g/kg/day, grams per kilogram of body weight per day; wk, week; wks, weeks; a.m., ante meridiem; p.m., post meridiem.

### Results of individual studies

Almeida et al. (33) found that creatine monohydrate supplementation did not significantly affect the lipid profile compared with placebo. Specifically, triglycerides, total cholesterol, LDL-C, and HDL-C were not significantly influenced by supplementation in either group. Overall, the findings indicated that short-term creatine supplementation did not produce clinically relevant or statistically significant alterations in blood lipid markers.

Almeida et al. (34) observed significant increases in body weight and one-repetition maximum (1RM) strength across all evaluated exercises in the creatine group compared to placebo. Regarding safety, although the creatine group showed statistically significant between-group differences in some blood markers (hematocrit, LDL-C, uric acid, alkaline phosphatase, and Creatinine), the changes were of small magnitude and all values remained within clinical reference ranges, indicating no evident health risk.

Arciero et al. (10) also reported that creatine supplementation, both with and without resistance training, led to significant increases in body mass and total body water. The combination of creatine and resistance training (Cr-RT) resulted in a significant increase in fat-free mass and produced greater strength gains in the leg press and bench press compared to creatine or resistance training alone. Additionally, the Cr-RT group experienced a significant increase in resting metabolic rate and calf and forearm blood flow, as well as a significant reduction in total cholesterol. No adverse effects on health were reported.

Clarke et al. (8) observed significant improvements in vascular function and metabolic markers following creatine monohydrate (CrM) supplementation in older adults. The study reported significant increases in macrovascular function, measured by flow-mediated dilation (FMD %), and in microvascular function, indicated by the tissue oxygen saturation (StO₂) reperfusion rate. Additionally, significant reductions in fasting glucose and triglyceride levels were documented. However, no significant changes were observed in arterial stiffness (velocity and pulse wave analysis), body fluid distribution, or oxidative stress biomarkers (malondialdehyde, oxidized LDL-C, and tetrahydrobiopterin), nor were there significant changes in SBP, DBP, or resting HR after CrM supplementation.

In contrast, Cornelissen et al. (19) found that creatine supplementation provided no additional benefit to cardiac patients undergoing a structured exercise training program. The study reported that both the creatine and placebo groups showed significant improvements in cardiorespiratory fitness (peak VO₂), muscle performance (peak torque and endurance), health-related quality of life, and specific blood lipids (increased HDL-C and decreased triglycerides) following the training intervention. However, the changes in these outcomes were similar between the creatine and placebo groups, showing no additive effect of creatine supplementation. Furthermore, no detrimental effects on renal or liver function were reported in either study, supporting the safety of creatine supplementation in these populations.

Earnest et al. (20) documented significant reductions in plasma total cholesterol, triacylglycerols (TAG), and very-low-density lipoprotein cholesterol (VLDL) following creatine monohydrate supplementation in hypercholesterolemia men and women. These lipid-lowering effects were observed at both 4 and 8 weeks of supplementation, with the reductions in TAG and VLDL persisting 4 weeks after supplementation ceased. A trend towards a reduction in fasting plasma glucose was also noted in males. However, no significant changes were observed in LDL-C, HDL-C, the total cholesterol/HDL-C ratio, or body composition measures.

In contrast, Gualano et al. (9) found that creatine supplementation provided no additional benefit to the lipid profile of healthy, sedentary males undergoing cardiorespiratory training. The study reported that both the creatine and placebo groups showed similar, significant improvements in HDL-C (increased) and TAG/VLDL (decreased) as a result of the training intervention. No between-group differences were observed for total cholesterol, LDL-C, fasting glucose, fasting insulin, or insulin resistance (HOMA index).

Conversely, Volek et al. (21) found that 12 weeks of heavy resistance training, with or without creatine supplementation, resulted in no significant changes in serum total cholesterol, HDL-C, LDL-C, or triglycerides in healthy, resistance-trained men, despite significant increases in body mass and fat-free mass.Synthesis of results

**Figure 2:**
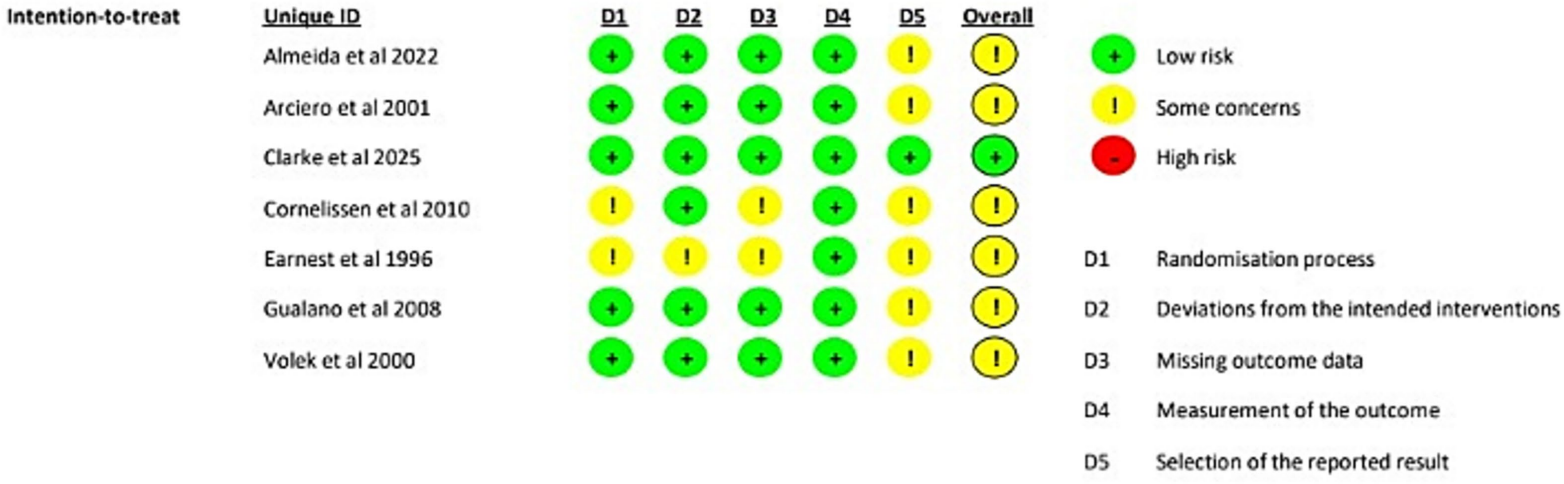
Meta-analysis for overall effects of creatine on total cholesterol, HDL cholesterol, LDL cholesterol and triglycerides.

The meta-analysis evaluated the impact of creatine supplementation on blood lipid profiles, specifically triglycerides, total cholesterol, HDL-C, and LDL-C, across multiple studies (Figure 2).

#### Total cholesterol

Seven randomized controlled trials (9, 10, 19–21, 33, 34) were included, comprising 111 participants in the creatine groups and 106 in the control groups. Individual study weights ranged from 5.7 to 28.8%, with Earnest et al. (20) contributing the largest weight.

Using a random-effects model, the pooled MD was 2.90 mg/dL (95% CI: −7.44 to 13.24). The overall effect was not statistically significant (*Z* = 0.55, *p* = 0.58). Moderate heterogeneity was observed (Tau^2^ = 78.06; Chi^2^ = 11.09, df = 6, *p* = 0.09; *I^2^* = 46%), indicating some variability across studies.

#### LDL-c

Five studies (9, 19, 21, 33, 34) contributed data, totaling 81 participants in the creatine groups and 82 in the control groups. Study weights ranged from 5.0 to 24.8%, with Almeida et al. (33) contributing the largest proportion of the pooled estimate.

Under a fixed-effect model, the pooled MD was 4.08 mg/dL (95% CI: −2.55 to 10.70). The overall effect was not statistically significant (*Z* = 1.21, *p* = 0.23). Low heterogeneity was detected (Chi^2^ = 4.34, df = 4, *p* = 0.36; *I^2^* = 8%), suggesting relative consistency among the included trials.

#### HDL-c

Five trials (9, 19, 21, 33, 34) were included, with 81 participants in the creatine groups and 82 in the control groups. Individual study weights ranged from 13.9 to 33.8%, with Cornelissen et al. (19) contributing the largest weight.

Using a fixed-effect model, the pooled MD was −0.68 mg/dL (95% CI: −3.94 to 2.59). The overall effect was not statistically significant (*Z* = 0.41, *p* = 0.68). No heterogeneity was observed (Chi^2^ = 2.89, df = 4, *p* = 0.58; *I^2^* = 0%), indicating high consistency across studies.

#### Triglycerides

Six studies (8, 9, 19, 21, 33, 34) were included, comprising 87 participants in the creatine groups and 88 in the control groups. Study weights ranged from 8.4 to 18.3%, with Cornelissen et al. (19) contributing the largest weight.

A random-effects model yielded a pooled MD of 7.95 mg/dL (95% CI: −13.73 to 29.63). The overall effect was not statistically significant (*Z* = 0.72, *p* = 0.47). Substantial heterogeneity was observed (Tau^2^ = 370.99; Chi^2^ = 11.17, df = 5, *p* = 0.05; *I^2^* = 55%), indicating considerable variability among the included studies.

#### Subgroup analysis

**Figure 3:**
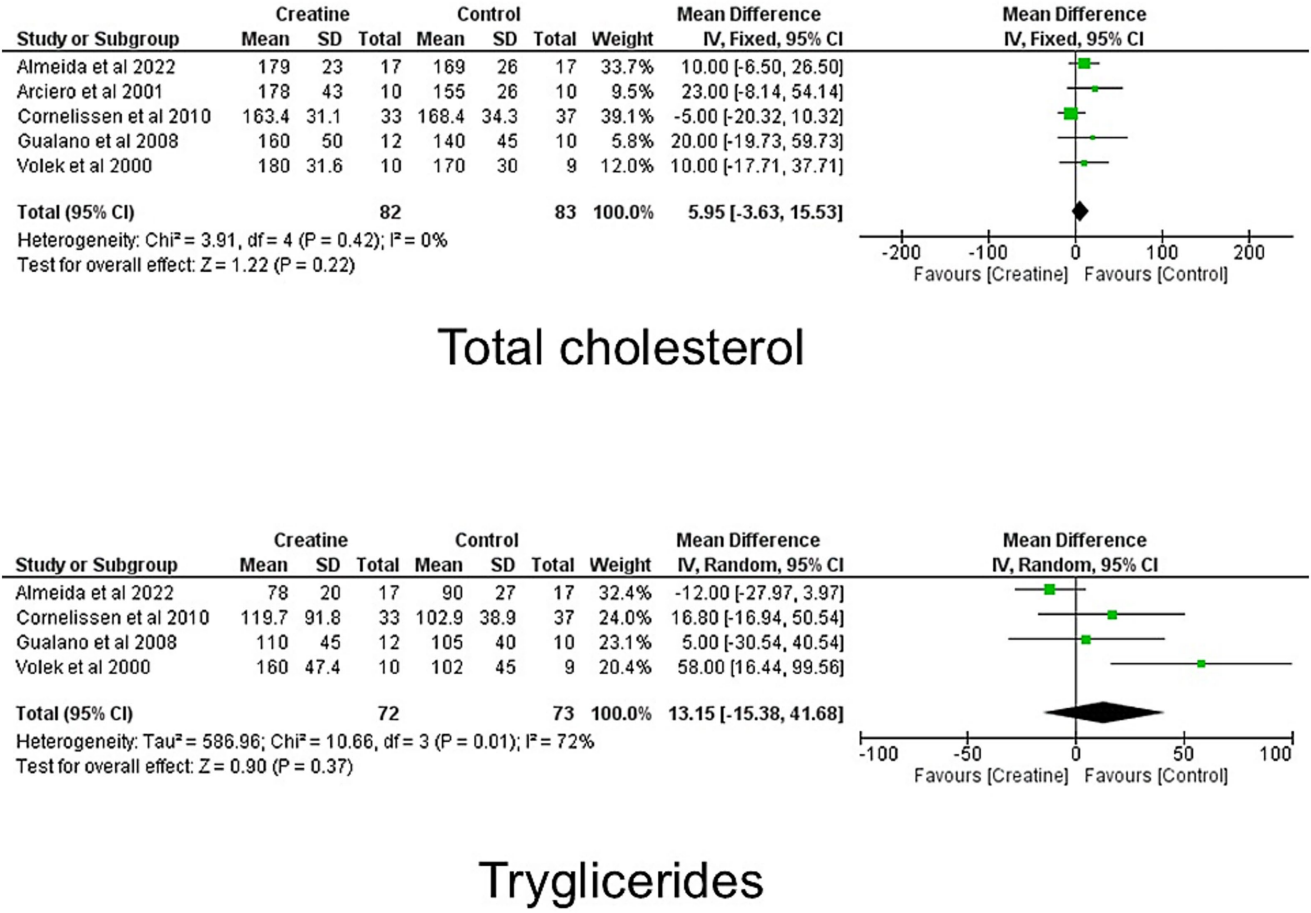
Cochrane risk of bias tool.

Subgroup analyses stratified according to the presence of concurrent exercise training were performed, and corresponding forest plots were generated to transparently present individual study effect sizes, 95% confidence intervals (CIs), study weights, pooled estimates, and heterogeneity statistics (Figure 3).

For total cholesterol, six studies (9, 10, 19, 21, 33, 34) were included, comprising 91 participants in the creatine groups and 92 in the control groups. Using a fixed-effect inverse-variance model, the pooled (MD was 6.43 mg/dL (95% CI: −2.72 to 15.58). The overall effect was not statistically significant (*Z* = 1.38, *p* = 0.17), indicating that creatine supplementation did not significantly affect total cholesterol compared with control. Individual study weights ranged from 5.3 to 35.7%, with Cornelissen et al. (19) contributing the greatest weight. No heterogeneity was detected across studies (Chi^2^ = 4.02, df = 5, *p* = 0.55; *I^2^* = 0%), suggesting high consistency among the included trials.

For triglycerides, four studies (9, 19, 33, 34) were included, totaling 71 participants in the creatine groups and 73 in the control groups. The pooled MD, also estimated using a fixed-effect inverse-variance model, was −4.51 mg/dL (95% CI: −17.60 to 8.59). This effect was not statistically significant (*Z* = 0.67, *p* = 0.50), indicating no significant difference between creatine and control for triglyceride levels. Study weights ranged from 4.1 to 67.2%, with Almeida et al. (34) providing the largest contribution to the pooled estimate. Heterogeneity was absent (Tau^2^ = 0.00; Chi^2^ = 2.81, df = 3, *p* = 0.42; *I^2^* = 0%), indicating that the effect estimates were highly consistent across studies.

Importantly, no formal assessment of publication bias was performed. Funnel plot asymmetry tests and regression-based methods (e.g., Egger’s test) are known to have very low statistical power and poor interpretability when the number of included studies is small. Methodological guidance indicates that such approaches are unreliable when fewer than 10 studies are available, as observed in the present analyses (k ranging from 3 to 5 per outcome) (16, 17). Under these conditions, visual inspection of funnel plots is also highly subjective and prone to misinterpretation. Therefore, conducting formal publication bias analyses would risk generating unstable and potentially misleading inferences.

Meta-regression was not conducted because reliable estimation of between-study variance and regression coefficients requires an adequate number of studies. Simulation and methodological work suggest that fewer than 10 studies per covariate yields imprecise and unstable estimates, with a high risk of type I and type II errors (16, 17). Given that the present meta-analyses included a maximum of five studies per outcome, any meta-regression model would have been statistically underpowered and methodologically inappropriate.

Accordingly, we prioritized reporting pooled effect estimates using random-effects models with transparent heterogeneity metrics (*I^2^* and *τ^2^*), while avoiding exploratory analyses that could overinterpret sparse data. This conservative approach minimizes the risk of unstable conclusions and aligns with established methodological recommendations for meta-analyses with a limited number of studies.

### Risk of bias

**Figure 4:**
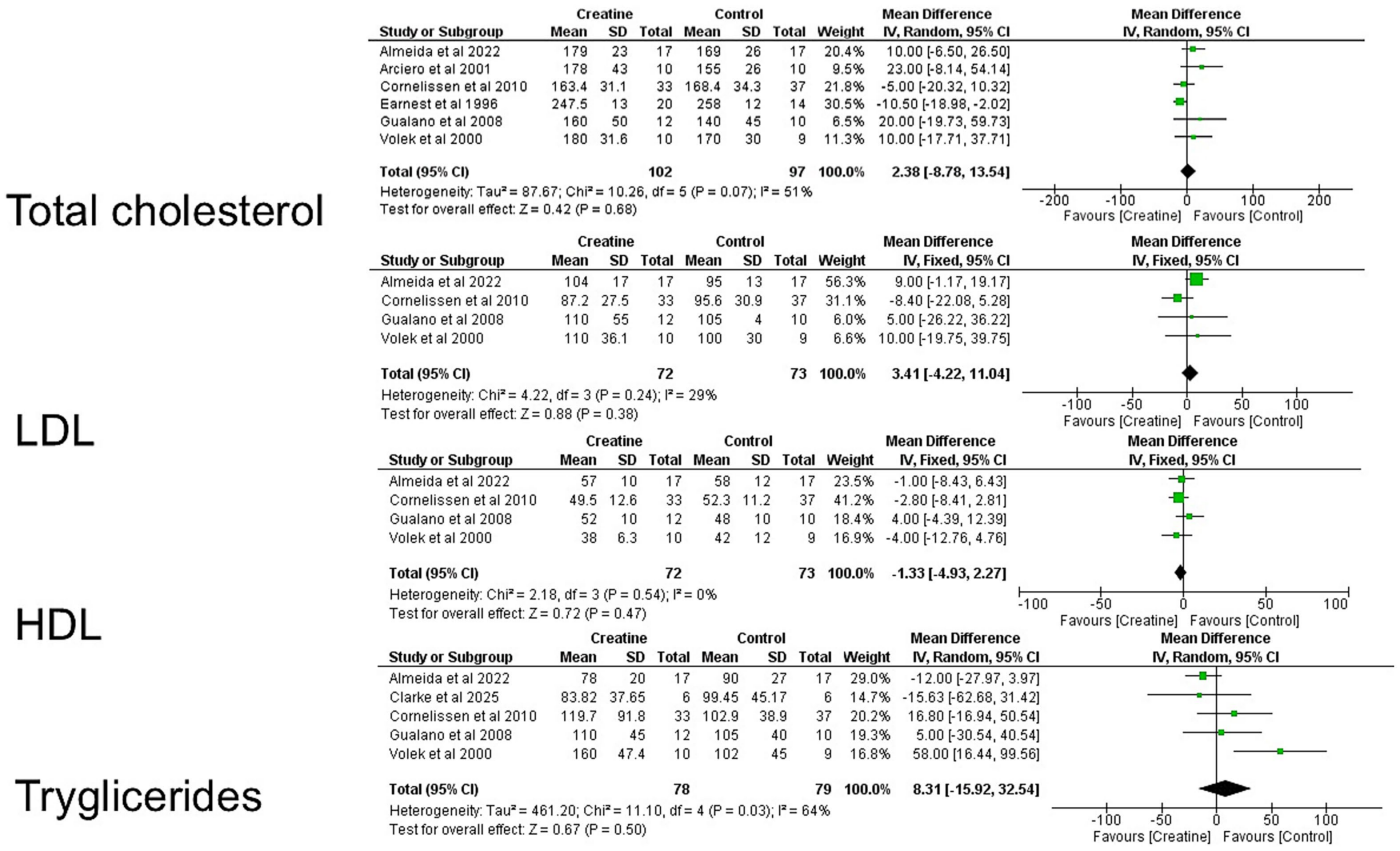
Forest plots comparing triglycerides, total cholesterol, HDL, and LDL between the creatine-using group and the control group.

The risk of bias for the included studies was assessed using the Cochrane RoB2 tool across five domains: the randomization process (D1), deviations from the intended interventions (D2), missing outcome data (D3), measurement of the outcome (D4), and selection of the reported result (D5), (Figure 4).

#### Randomization process (D1)

For the randomization process, most studies were judged to have a low risk of bias, including Almeida et al. (34), Arciero et al. (10), Clarke et al. (8), Gualano et al. (9), and Volek et al. (21). In contrast, Cornelissen et al. (19), Earnest et al. (20), and Almeida et al. (33) raised some concerns in this domain, suggesting possible limitations in the reporting or conduct of the randomization procedures.

#### Deviations from intended interventions (D2)

With respect to deviations from intended interventions, nearly all studies were judged to have a low risk of bias. The only exception was Earnest et al. (20), which raised some concerns in this domain. Overall, the evidence suggests that, in most trials, deviations from the assigned interventions were unlikely to have materially influenced the results.

#### Missing outcome data (D3)

For missing outcome data, the majority of studies demonstrated a low risk of bias, including Almeida et al. (34), Arciero et al. (10), Clarke et al. (8), Gualano et al. (9), Volek et al. (21), and Almeida et al. (33). However, Cornelissen et al. (19) and Earnest et al. (20) raised some concerns, indicating potential issues related to incomplete outcome data or insufficient reporting on attrition.

#### Measurement of the outcome (D4)

In the domain of outcome measurement, all included studies were judged to have a low risk of bias. This indicates that outcome assessment methods were considered appropriate and were unlikely to have introduced systematic error across the trials.

#### Selection of the reported result (D5)

The selection of the reported result was the domain with the greatest frequency of concerns. Only Clarke et al. (8) was judged to have a low risk of bias in this domain. All other studies, Almeida et al. (34), Arciero et al. (10), Cornelissen et al. (19), Earnest et al. (20), Gualano et al. (9), Volek et al. (21), and Almeida et al. (33), raised some concerns, possibly reflecting limited information regarding prespecified analyses or selective outcome reporting.

#### Overall bias

Overall, Clarke et al. (8) was the only study judged to have a low risk of bias across all domains. The remaining studies were classified as having some concerns overall, largely driven by issues in the selection of the reported result and, in some cases, by additional concerns related to randomization and missing outcome data. Specifically, Cornelissen et al. (19) presented some concerns in the domains of the randomization process (D1), missing outcome data (D3), and selection of the reported result (D5), whereas Earnest et al. (20) raised some concerns in deviations from intended interventions (D2) in addition to D1, D3, and D5. Almeida et al. (33) also raised some concerns overall, primarily due to issues in the randomization process (D1) and selection of the reported result (D5). No study was judged to have a high risk of bias in the overall assessment.

### GRADE assessment

The GRADE assessment demonstrated variability in the certainty of evidence across lipid-related outcomes following creatine supplementation, as detailed in Table 2. Triglycerides (six studies) were supported by very low-certainty evidence. Although the risk of bias was judged as not serious, the evidence was downgraded due to very serious inconsistency (*I^2^* = 55%), as well as serious indirectness related to differences in population characteristics and supplementation doses, and serious imprecision, reflecting the coexistence of positive and null effects within moderately wide 95% confidence intervals.

**Table 2:** Levels of evidence analysis via (GRADE working group, 2004).

Outcome	No. of studies	Risk of bias	Inconsistency	Indirectness	Imprecision	Certainty of evidence
Triglycerides	6	Not serious	Serious^a^	Serious^b^	Serious^c^	Very low
Total cholesterol	7	Not serious	Not serious	Serious^b^	Serious^c^	Very low
HDL-C	5	Not serious	Not serious	Serious^b^	Serious^c^	Low
LDL-C	5	Not serious	Not serious	Serious^b^	Serious^c^	Low

^a^*I^2^* = 55%; ^b^ different population and dose; ^c^ presence and absence of effect based on moderate 95% CI.

Similarly, total cholesterol (seven studies) was rated as having very low-certainty evidence. While risk of bias was not considered serious, the certainty was downgraded for serious indirectness stemming from heterogeneity in participant profiles and dosing regimens, and serious imprecision due to mixed findings across trials.

In contrast, HDL-C (five studies) and LDL-C (five studies) were both graded as providing low-certainty evidence. For these outcomes, neither risk of bias nor inconsistency was deemed serious. However, downgrading occurred due to serious indirectness, again attributable to variability in study populations and creatine doses, and serious imprecision, driven by the presence and absence of statistically significant effects across studies.

Overall, these findings indicate that the current body of evidence supporting the effects of creatine supplementation on HDL-C and LDL-C is slightly more robust than for triglycerides and total cholesterol; nevertheless, important methodological and clinical heterogeneity limits the overall strength and confidence of the conclusions.

### Heterogeneity

The serious inconsistency noted for the triglyceride and total cholesterol outcomes (*I^2^* = 55 and 46%, respectively) may be explained by clinical and methodological diversity between the included studies. While the populations across studies were broadly similar (e.g., healthy, athletic, or older adults), there were differences in supplementation protocols (dose and duration), the type of concurrent exercise training (e.g., resistance vs. cardiorespiratory exercise), and the baseline health and training status of the participants. This variability likely contributed to the substantial heterogeneity observed in the effect of creatine on total cholesterol and triglyceride levels. In contrast, the evidence for HDL-C and LDL-C was consistent across studies, contributing to the high certainty ratings.

## Discussion

Our systematic review and meta-analysis aimed to evaluate the effects of creatine supplementation on blood lipid profiles. Our main findings are:

1. The meta-analysis showed no statistically significant effect of creatine supplementation on total cholesterol, HDL-C, LDL-C, or triglycerides. Subgroup meta-analyses stratified by the presence of concurrent exercise training (creatine plus exercise vs. creatine alone) were performed to explore heterogeneity; however, results remained non-significant across lipid outcomes. For triglycerides, substantial heterogeneity persisted, and subgroup analysis did not meaningfully modify the findings, indicating that exercise co-intervention alone does not explain the observed variability.
2. The quality of evidence, assessed via GRADE, demonstrated very low certainty for total cholesterol and triglycerides, and low certainty for HDL-C, and LDL-C outcomes.
3. The risk of bias analysis highlighted recurring concerns regarding the selection of the reported result (D5) across most studies, while the randomization process (D1) and missing outcome data (D3) raised concerns in several instances.

It was previously emphasized that the renewed interest in creatine biology, with recent discoveries pointing to a diverse range of regulatory functions performed by this compound due to technological advances in genetic engineering and metabolism, coupled with the understanding that this metabolite exerts significant functions in cells beyond muscle and brain tissue. According to them, specifically in adipose tissue, creatine regulates thermogenic respiration, and its deficiency compromises systemic energy expenditure, potentially culminating in obesity. Additionally, creatine metabolism influences the survival of neoplastic cells and the functionality of the immune system (22).

With this in mind, preclinical studies remain essential for elucidating mechanistic pathways and biological plausibility, as demonstrated by experimental models showing exercise-induced modulation of hyperalgesia and cardiomyocyte structural preservation under stress (23, 24). This broader perspective is supported by preclinical research reporting favorable metabolic effects. Chen et al. (25) examined the ability of creatine supplementation to mitigate obesity caused by a high-fat diet in mice. The authors found that creatine promoted significant improvements in metabolic health, reversing body mass gain, increasing insulin sensitivity, and decreasing lipid accumulation in the liver. A key mechanism identified was the activation of brown adipose tissue, a type of fat specialized in energy dissipation, and the regulation of two central processes in lipid degradation: lipolysis and lipophagy, in both brown adipose tissue and the liver. In summary, the study proposes that creatine acts as a brown adipose tissue activator, enhancing global energy metabolism by coordinating these lipid catabolism mechanisms.

Evidence from other animal species corroborates this modulating potential as described by Hu et al (2025). In juvenile grass carp, dietary creatine supplementation significantly improved the impairments in growth performance and feed efficiency induced by a high-fat diet. Additionally, dietary creatine reduced liver lipid accumulation caused by high-fat diet by stimulating fatty acid *β*-oxidation, a process mediated by mitochondrial fusion dependent on the Mfn2 protein. Notably, this research elucidated a novel molecular mechanism, demonstrating that creatine induces mitochondrial fusion by binding transcription factors to specific sites in the mitofusin 2 (Mfn2) gene promoter (26).

Beyond its role in energy metabolism and adipose tissue regulation, emerging clinical evidence suggests that creatine may exert cardiovascular protection through peripheral and endothelial mechanisms independent of direct lipid lowering. Recent work has highlighted creatine’s antioxidant and vasoprotective properties, which may influence vascular function even in the absence of significant changes in serum cholesterol (27, 28).

Clarke et al. (28) proposed that creatine may enhance vascular health by reducing oxidative stress and improving nitric oxide bioavailability. They mentioned that creatine appears to modulate reactive oxygen species production through mitochondrial creatine kinase–dependent mechanisms, thereby preserving mitochondrial energetics and limiting endothelial dysfunction. In experimental and human models, creatine has demonstrated anti-inflammatory effects, including reduced expression of endothelial adhesion molecules and attenuation of oxidative damage, processes that are closely linked to atherosclerotic progression and impaired vascular reactivity (28). These mechanisms provide a biologically plausible explanation for cardiovascular benefits that are not necessarily mediated by changes in LDL-C or HDL-C concentrations.

In addition, more recently, Aron et al. (27) conducted a randomized controlled trial in older men and reported that acute creatine supplementation improved vascular stiffness, as evidenced by reductions in the cardio-ankle vascular index. Although traditional hemodynamic variables showed only trends toward improvement, the significant enhancement in arterial stiffness suggests that creatine may positively influence macrovascular function. Importantly, these effects occurred over a short supplementation period and were independent of lipid profile modifications.

Taken together, these findings indicate that creatine’s cardiovascular impact may extend beyond classical lipid modulation. While our meta-analysis did not demonstrate consistent reductions in total cholesterol, LDL-C, HDL-C, or triglycerides, creatine may confer vascular benefits through improvements in endothelial function, oxidative balance, and arterial compliance. This broader physiological perspective strengthens the interpretation that absence of lipid changes does not preclude potential cardioprotective effects.

However, clinical studies in humans have yielded less conclusive results (29). Gualano et al. (9) investigated the effects of creatine (Cr) supplementation on the plasma lipid profile of sedentary men undergoing a cardiorespiratory training program. Cr supplementation had no additional effect on the lipid profile of healthy individuals after 3 months of moderate-intensity cardiorespiratory training, as significant temporal effects were observed in both groups for HDL-C, triglycerides, and VLDL. However, no intergroup differences were detected in HDL-C, LDL-C, TC, VLDL, or triglyceride levels. Furthermore, parameters such as fasting insulin and glucose, as well as the homeostasis model assessment index (HOMA), did not undergo significant changes.

This lack of significant effect in humans is further supported by comprehensive analyses. Our quantitative analysis did not confirm a significant beneficial impact of creatine supplementation on blood lipid profiles. Data from selected references [(8, 9, 19, 21, 34), etc.] allowed us to conduct a meta-analysis for triglycerides, total cholesterol, HDL-C, and LDL-C, important biomarkers for cardiovascular risk. Regarding triglycerides, the creatine-supplemented group showed a mean difference compared to the placebo group. This result, however, did not reach statistical significance (*p* = 0.37). Considerable heterogeneity was recorded among the included studies (*I^2^* = 55%, *p* = 0.005), indicating substantial variation in their individual results. Importantly, the lack of a statistically significant effect of creatine supplementation persisted even after conducting subgroup analyses stratified by the presence of concurrent exercise training.

The findings of our meta-analysis suggest that creatine supplementation does not produce consistent or clinically meaningful improvements in the lipid profile. The pooled estimates for the outcomes showed no indication of benefit, which is likely due to the high degree of biological stability of these markers and the limited capacity of short-term creatine interventions to influence lipoprotein metabolism in humans. In contrast, the triglycerides outcome exhibited substantial heterogeneity, indicating considerable variability among studies. This inconsistency may be explained by differences in supplementation protocols, baseline metabolic risk, physical activity levels, and concurrent training programs, all of which could independently modulate triglyceride responses. Together, these findings suggest that any potential lipid-modifying effect of creatine is modest at best and highly dependent on study-specific factors, underscoring the need for more methodologically homogeneous trials to clarify these associations.

Based on these data, it is inferred that there is no robust evidence that creatine supplementation produces significant improvements or deleterious effects on serum triglyceride, total cholesterol, HDL-C, and LDL-C levels in humans when compared to animal studies, in which creatine demonstrates the ability to modulate hepatic lipid metabolism and adipose tissue. This disparity may be attributed to interspecies differences, specific pathological contexts versus health conditions, or uncontrolled methodological variables. Therefore, although the therapeutic potential of creatine warrants future investigation, especially in populations with established metabolic disorders, current evidence does not support creatine supplementation as an effective strategy for improving lipid profiles in the general human population.

In addition to the absence of lipid-modifying effects observed in the present meta-analysis, the overall safety profile of creatine supplementation should also be considered. A comprehensive analysis conducted by Kreider et al. (30) evaluated reported side effects across 685 human clinical trials, including more than 12,800 participants receiving creatine and over 13,400 receiving placebo. The prevalence of studies reporting side effects was similar between groups (13.7% in creatine groups vs. 13.2% in placebo groups), with no statistically significant difference. When the analysis focused on individual participants, 4.60% of those consuming creatine reported any side effect compared with 4.21% in placebo groups, again without significant differences.

Multivariate analyses examining 49 potential adverse effects revealed no meaningful increase in the frequency of side effects attributable to creatine supplementation. Importantly, the analysis also found no consistent evidence of increased renal, hepatic, cardiovascular, or metabolic adverse events associated with creatine use. Beyond clinical trials, the authors also examined pharmacovigilance data from large international adverse event reporting systems. Among more than 28.4 million adverse event reports, only 203 mentioned creatine-containing products, corresponding to approximately 0.00072% of all reports. Moreover, many of these cases involved multi-ingredient supplements or concurrent medication use, making direct attribution to creatine alone unlikely.

Taken together, the abovementioned findings indicate that creatine supplementation is well tolerated and not associated with an increased prevalence or frequency of clinically significant adverse events when compared with placebo. These results reinforce the broader scientific consensus that creatine monohydrate is a safe nutritional supplement when consumed within commonly recommended dosages.

Regarding the limitations of the primary studies and the meta-analysis, issues that directly impact the robustness and interpretation of the results stand out. First, the meta-analyses for total cholesterol and triglycerides were marked by high heterogeneity. This fact indicates considerable variability in the methodology, study populations, and interventions implemented by the different studies included. This heterogeneity can be attributed to differences in exercise protocols, dosage and duration of creatine supplementation, the basal metabolic state of the participants, and their dietary habits. This diversity makes it difficult to draw a single, generalizable conclusion.

Moreover, the risk of bias analysis revealed significant concerns in critical areas. Some studies presented concerns in the domain of selection of reported results, which raises the possibility of reporting bias, where only statistically significant results were reported. Additionally, in several cases, there were concerns regarding the randomization process, which is a cornerstone of the internal validity of a clinical trial. Failures in these fundamental processes may have introduced systematic biases, compromising the reliability of the results aggregated by the meta-analysis.

These methodological weaknesses also extend to issues related to missing outcome data, which can further distort the pooled estimates if losses to follow-up differ between intervention and control groups or if incomplete data disproportionately affect specific lipid outcomes. In several of the included trials, insufficient clarity about how missing data were handled, whether via complete-case analysis, last observation carried forward, or unreported imputation, introduces uncertainty regarding the accuracy of the reported effect sizes. Likewise, concerns about selective reporting suggest that some outcomes may have been measured but not fully disclosed, especially when nonsignificant or unfavorable. This type of bias can inflate or obscure true effects by over representing studies with positive findings and underreporting null results.

The broad temporal span of the included studies, ranging from 1996 to 2025, is an important factor to consider. Over these nearly three decades, significant progress has been made in lipid quantification techniques, including enhanced assay sensitivity, improved calibration standards, and stricter laboratory quality control procedures (31, 32). Earlier studies might have relied on less standardized enzymatic assays, manual processing, or lab-specific reference ranges. In contrast, more recent trials typically utilize automated platforms that offer greater precision, traceability to international reference standards, and robust external quality assessment programs. This methodological evolution may introduce variability in measured lipid concentrations independent of the intervention itself.

While most clinical laboratories follow standardized protocols, subtle temporal variations in analytical performance characteristics could contribute to heterogeneity between studies, especially for triglycerides, which are particularly susceptible to pre-analytical and analytical variation. Due to the lack of individual participant data, it was impossible to formally adjust for the laboratory era or assay methodology in the pooled analyses. Consequently, these temporal differences in lipid detection technologies represent a potential, unaddressed confounding factor that may partially account for the variability observed across trials and should be taken into account when interpreting the results.

An additional limitation relates to the inability to retrieve the full text of one potentially eligible study, which was therefore classified as “not retrieved” in the PRISMA flow diagram. Despite repeated attempts, including direct contact with the corresponding author and efforts to obtain temporary paid access through the publisher’s website, the complete manuscript could not be accessed. Consequently, it was not possible to verify detailed methodological information or extract complete lipid outcome data for quantitative synthesis. To preserve data accuracy and methodological transparency, we refrained from including partially verified or secondary data. Although only one study was affected, its exclusion may have slightly reduced the overall sample size and precision of pooled estimates, representing an unavoidable but relevant limitation of the present review.

Taken together, these risks of bias highlight important limitations in the primary literature and may partially explain the variability observed in the meta-analysis. Strengthening future trials with transparent randomization procedures, predefined statistical analysis plans, and comprehensive reporting of all measured outcomes will be essential to improve the certainty and interpretability of evidence on creatine’s effects on lipid metabolism.

Within this context, the GRADE assessment offers essential insight into the level of confidence that can be placed in the findings. For total cholesterol, HDL-C, and LDL-C, the certainty of evidence was rated as low, reflecting that although these outcomes showed no serious concerns related to risk of bias or inconsistency, the evidence was downgraded due to serious indirectness, stemming from differences in study populations and dosing strategies, and serious imprecision, given the presence and absence of effects across trials. These limitations indicate that, while the overall patterns across studies appear relatively stable, the strength of the conclusions regarding creatine’s influence on these lipid markers remains limited.

By contrast, the certainty of evidence for triglycerides was rated as very low, primarily because of very serious inconsistency, as demonstrated by substantial heterogeneity (*I^2^* = 55%) in the pooled analysis. This high degree of variability suggests that the included studies differed markedly in both the direction and magnitude of their effects. Such discrepancies imply that triglyceride responses to creatine may be influenced by factors such as baseline metabolic characteristics, training status, variations in supplementation protocols, or lifestyle behaviors that were not consistently controlled across trials.

The differing certainty ratings carry important implications for clinical interpretation. The low-certainty evidence for total cholesterol, HDL-C, and LDL-C indicates that, although current findings suggest creatine supplementation does not meaningfully affect these lipid markers, the overall confidence in this conclusion is limited due to concerns related to indirectness and imprecision. As a result, clinicians and researchers should interpret these outcomes cautiously, recognizing that the true effects may differ from the summarized estimates.

In contrast, the very low-certainty evidence for triglycerides signals an even greater degree of uncertainty. The very serious inconsistency observed across studies, reflected in substantial heterogeneity, suggests that the actual effect of creatine on triglyceride levels could vary widely depending on population characteristics, dosing strategies, or lifestyle factors not uniformly controlled. Although the available data do not support a triglyceride-lowering effect, the pronounced variability underscores the need for better-designed and more homogeneous trials before firm conclusions can be made.

Taken together, the GRADE findings indicate that creatine should not be viewed as a lipid-modifying supplement and highlight important gaps in the literature where further research is clearly needed.

The identified limitations, notably heterogeneity and risks of bias, emphasize the need for future studies with more rigorous methodological designs, including pre-registered randomization and analysis protocols and with more homogeneous populations, to definitively clarify the role of creatine in lipid metabolism in humans.

Future randomized controlled trials should aim to overcome current methodological limitations by implementing more rigorous study designs with larger and more diverse populations. Special attention should be given to standardizing creatine supplementation protocols, including dosage, duration, and formulation, as well as controlling for confounding factors such as physical activity levels and dietary habits. Additionally, future studies should prioritize populations at higher cardiometabolic risk, such as individuals with obesity, type 2 diabetes, or dyslipidemia, where the potential impact of creatine on lipid metabolism may be more pronounced. The inclusion of mechanistic endpoints, such as biomarkers of lipogenesis, fatty acid oxidation, and mitochondrial function, could provide valuable insights into the underlying physiological pathways. Moreover, pre-registered protocols, double-blind placebo-controlled designs, and long-term follow-ups are essential to improve internal validity and assess the sustainability of any observed effects. Ultimately, a more precise characterization of creatine’s systemic effects could pave the way for its potential integration into metabolic health interventions beyond athletic contexts.

## Conclusion

This systematic review and meta-analysis indicated that creatine supplementation does not significantly affect the lipid profile in humans, demonstrating no clinically relevant effects on total cholesterol, HDL-C, LDL-C, or triglyceride levels. Furthermore, due to the substantial heterogeneity observed in some outcomes, the low to very low certainty of evidence, and the risks of bias identified in critical methodological domains, we suggest conducting future studies with robust, double-blind, placebo-controlled designs to provide more robust scientific and statistical evidence on this topic.

## Data Availability

The original contributions presented in the study are included in the article/supplementary material, further inquiries can be directed to the corresponding author.
